# Supplementary material for: The Definition, Assessment, and Prevalence of (Human Assumed) Central Sensitisation in Patients with Chronic Low Back Pain: A Systematic Review
Source: J Clin Med. 2021 Dec 17;10(24):5931. doi: 10.3390/jcm10245931 (PMC8703986; doi:10.3390/jcm10245931)
Supplement: Supplementary file 1 [file jcm-10-05931-s001.zip › jcm-1454927-supplementary.pdf]

Suppenmentary Metarials

File S1. Search Strategy

MEDLINE

("Central Nervous System Sensitisation" [Mesh] OR "Somatosensory Disorders" [Mesh] OR Central Sensitisation* [tiab] OR Central Sensitisation* [tiab] OR Pain sensit* [tiab] OR Central nervous system sensitisation* [tiab] OR Central nervous system sensitisation* [tiab] OR Central hypersensitiv* [tiab] OR Somatosensory disorder* [tiab] OR Pain hypersensitiv* [tiab] OR Neuropathic sensitisation* [tiab] OR Neuropathic sensitisation* [tiab] OR Perceptual sensitisation* [tiab] OR Perceptual sensitisation* [tiab] OR Neural sensitisation* [tiab] OR Neural sensitisation* [tiab] OR Central sensitivity syndrome* [tiab] OR Allodynia [tiab] OR Hyperalgesia [tiab] OR Hyperesthesia [tiab] OR Hyperpathia [tiab] OR Paresthesia [tiab] OR Dysesthesia [tiab] OR Analgesia [tiab] OR Hypoalgesia [tiab] OR Hypoesthesia [tiab])

AND

("Low Back Pain" [Mesh] OR "Lumbosacral Region" [Mesh] OR "Lumbar vertebrae" [Mesh] OR "Radiculopathy" [Mesh] OR "sciatica" [Mesh] OR "Pain, Referred" [Mesh] OR "Chronic Pain" [Mesh] OR Low back pain* [tiab] OR Lower back pain* [tiab] OR Lumbosacral region* [tiab] OR Lumbar vertebra* [tiab] OR Radiculopathy [tiab] OR Sciatica [tiab] OR Leg pain [tiab] OR Leg radiation* [tiab] OR Referred pain* [tiab] OR Radiating pain* [tiab] OR Radicular pain* [tiab] OR Pseudoradicular pain* [tiab] OR Lumbosacral radicular syndrome* [tiab] OR Chronic low back pain* [tiab] OR chronic back pain* [tiab] OR chronic pain* [tiab])

AND

(instrumentation [sh] OR methods [sh] OR "Validation Study" [pt] OR "Comparative Study" [pt] OR "psychometrics" [MeSH] OR psychometr* [tiab] OR clinimetr* [tiab] OR clinometr* [tiab] OR "outcome assessment, health care" [MeSH] OR "outcome assessment" [tiab] OR "outcome measure*" [tiab] OR "observer variation" [MeSH] OR "observer variation" [tiab] OR "Health Status Indicators" [Mesh] OR "reproducibility of results" [MeSH] OR reproducib* [tiab] OR "discriminant analysis" [MeSH] OR reliab* [tiab] OR unreliab* [tiab] OR valid* [tiab] OR "coefficient of variation" [tiab] OR coefficient [tiab] OR homogeneity [tiab] OR homogeneous [tiab] OR "internal consistency" [tiab] OR (cronbach* [tiab] AND (alpha [tiab] OR alphas [tiab])) OR (item [tiab] AND (correlation* [tiab] OR selection* [tiab] OR reduction* [tiab])) OR agreement [tiab] OR precision [tiab] OR imprecision [tiab] OR "precise values" [tiab] OR test-retest [tiab] OR (test [tiab] AND retest [tiab]) OR (reliab* [tiab] AND (test [tiab] OR retest [tiab])) OR stability [tiab] OR interrater [tiab] OR inter-rater [tiab] OR intrarater [tiab] OR intra-rater [tiab] OR intertester [tiab] OR inter-tester [tiab] OR intratester [tiab] OR intra-tester [tiab] OR interobserver [tiab] OR inter-observer [tiab] OR intraobserver [tiab] OR intra-observer [tiab] OR intertechnician [tiab] OR inter-technician [tiab] OR intratechnician [tiab] OR intra-technician [tiab] OR interexaminer [tiab] OR inter-examiner [tiab] OR intraexaminer [tiab] OR intra-examiner [tiab] OR interassay [tiab] OR inter-assay [tiab] OR intraassay [tiab] OR intra-assay [tiab] OR interindividual [tiab] OR inter-individual [tiab] OR intraindividual [tiab] OR intra-individual [tiab] OR interparticipant [tiab] OR inter-participant [tiab] OR intraparticipant [tiab] OR intra-participant [tiab] OR kappa [tiab] OR kappa's [tiab] OR kappas [tiab] OR repeatab* [tiab] OR ((replicab* [tiab] OR repeated [tiab]) AND (measure [tiab] OR measures [tiab] OR findings [tiab] OR result [tiab] OR results [tiab] OR test [tiab] OR tests [tiab])) OR generaliza* [tiab] OR generalisa* [tiab] OR concordance [tiab] OR (intraclass [tiab] AND correlation* [tiab]) OR discriminative [tiab] OR "known group" [tiab] OR "factor analysis" [tiab] OR "factor analyses" [tiab] OR "factor structure" [tiab] OR "factor structures" [tiab] OR dimension* [tiab] OR subscale*

[tiab] OR (multitrait [tiab] AND scaling [tiab] AND (analysis [tiab] OR analyses [tiab])) OR “item discriminant” [tiab] OR “interscale correlation*” [tiab] OR error [tiab] OR errors [tiab] OR “individual variability” [tiab] OR “interval variability” [tiab] OR “rate variability” [tiab] OR (variability [tiab] AND (analysis [tiab] OR values [tiab])) OR (uncertainty [tiab] AND (measurement [tiab] OR measuring [tiab])) OR “standard error of measurement” [tiab] OR sensitiv* [tiab] OR responsive* [tiab] OR (limit [tiab] AND detection [tiab]) OR “minimal detectable concentration” [tiab] OR interpretab* [tiab] OR ((minimal [tiab] OR minimally [tiab] OR clinical [tiab] OR clinically [tiab]) AND (important [tiab] OR significant [tiab] OR detectable [tiab]) AND (change [tiab] OR difference [tiab])) OR (small* [tiab] AND (real [tiab] OR detectable [tiab]) AND (change [tiab] OR difference [tiab])) OR “meaningful change” [tiab] OR “ceiling effect” [tiab] OR “floor effect” [tiab] OR “Item response model” [tiab] OR IRT [tiab] OR Rasch [tiab] OR “Differential item functioning” [tiab] OR DIF [tiab] OR “computer adaptive testing” [tiab] OR “item bank” [tiab] OR “cross-cultural equivalence” [tiab])

NOT

(“Animals” [Mesh] NOT “Humans” [Mesh])

EMBASE

(‘central nervous system sensitisation’/exp OR ‘somatosensory disorder’/exp OR (“central sensitisation*” OR “central sensitisation*” OR “pain sensit*” OR “central nervous system sensitisation*” OR “central nervous system sensitisation*” OR “central hypersensitiv*” OR “somatosensory disorder*” OR “pain hypersensitiv*” OR “neuropathic sensitisation*” OR “neuropathic sensitisation*” OR “perceptual sensitisation*” OR “perceptual sensitisation*” OR “neural sensitisation*” OR “neural sensitisation*” OR “central sensitivity syndrome*” OR “Allodynia” OR “Hyperalgesia” OR “Hyperesthesia” OR “Hyperpathia” OR “Paresthesia” OR “Dysesthesia” OR ”Analgesia” OR ”Hypoalgesia” OR ”Hypoesthesia”):ab,ti)

AND

(‘low back pain*’/exp OR ’lumbosacral region*’/exp OR ‘lumbar vertebra*’/exp OR ‘radiculopathy’/exp OR ‘sciatica’/exp OR ‘referred pain*’/exp OR ‘chronic pain*’/exp OR (“low back pain*” OR “lower back pain*” OR “lumbosacral region*” OR “lumbar vertebra*” OR “radiculopathy” OR “sciatica” OR “leg pain*” OR “leg radiation*” OR “referred pain*” OR “radiating pain*” OR “radicular pain*” OR “pseudoradicular pain*” OR “lumbosacral radicular syndrome*” OR “chronic low back pain*” OR “chronic pain*” OR “chronic back pain*”):ab,ti)

AND

(‘intermethod comparison’/exp OR ‘data collection method’/exp OR ‘validation study’/exp OR ‘feasibility study’/exp OR ‘pilot study’/exp OR ‘psychometry’/exp OR ‘reproducibility’/exp OR reproducib*:ab,ti OR ‘audit’:ab,ti OR psychometr*:ab,ti OR clinimetr*:ab,ti OR clinometr*:ab,ti OR ‘observer variation’/exp OR ‘observer variation’:ab,ti OR ‘discriminant analysis’/exp OR ‘validity’/exp OR reliab*:ab,ti OR valid*:ab,ti OR ‘coefficient’:ab,ti OR ‘internal consistency’:ab,ti OR (cronbach*:ab,ti AND (‘alpha’:ab,ti OR ‘alphas’:ab,ti)) OR ‘item correlation’:ab,ti OR ‘item correlations’:ab,ti OR ‘item selection’:ab,ti OR ‘item selections’:ab,ti OR ‘item reduction’:ab,ti OR ‘item reductions’:ab,ti OR ‘agreement’:ab,ti OR ‘precision’:ab,ti OR ‘imprecision’:ab,ti OR ‘precise values’:ab,ti OR ‘test-retest’:ab,ti OR (‘test’:ab,ti AND ‘retest’:ab,ti) OR (reliab*:ab,ti AND (‘test’:ab,ti OR ‘retest’:ab,ti)) OR ‘stability’:ab,ti OR ‘interrater’:ab,ti OR ‘inter-rater’:ab,ti OR ‘intrarater’:ab,ti OR ‘intra-rater’:ab,ti OR ‘intertester’:ab,ti OR ‘inter-tester’:ab,ti OR ‘intratester’:ab,ti OR ‘intratester’:ab,ti OR ‘interobeserver’:ab,ti OR ‘inter-observer’:ab,ti OR ‘intraobserver’:ab,ti OR ‘intraobserver’:ab,ti OR ‘intertechnician’:ab,ti OR ‘inter-technician’:ab,ti OR ‘intratechnician’:ab,ti OR ‘intratechnician’:ab,ti OR ‘interexaminer’:ab,ti OR ‘inter-examiner’:ab,ti OR ‘intraexaminer’:ab,ti OR ‘intraexaminer’:ab,ti OR ‘interassay’:ab,ti OR ‘inter-assay’:ab,ti OR ‘intraassay’:ab,ti OR ‘intra-assay’:ab,ti OR ‘interindividual’:ab,ti OR ‘inter-individual’:ab,ti OR ‘intraindividual’:ab,ti OR ‘intra-individual’:ab,ti OR ‘interparticipant’:ab,ti OR ‘inter-participant’:ab,ti OR ‘intraparticipant’:ab,ti OR ‘intraparticipant’:ab,ti OR ‘kappa’:ab,ti OR ‘kappas’:ab,ti OR ‘coefficient of variation’:ab,ti OR repeatab*:ab,ti OR (replicab*:ab,ti OR ‘repeated’:ab,ti AND (‘measure’:ab,ti OR ‘measures’:ab,ti OR ‘findings’:ab,ti OR ‘result’:ab,ti OR ‘results’:ab,ti OR ‘test’:ab,ti OR ‘tests’:ab,ti)) OR generaliza*:ab,ti OR generalisa*:ab,ti OR ‘concordance’:ab,ti OR (‘intraclass’:ab,ti AND correlation*:ab,ti) OR ‘discriminative’:ab,ti OR ‘known group’:ab,ti OR ‘factor analysis’:ab,ti OR ‘factor analyses’:ab,ti OR ‘factor structure’:ab,ti OR ‘factor structures’:ab,ti OR ‘dimensionality’:ab,ti OR subscale*:ab,ti OR ‘multitrait scaling analysis’:ab,ti OR ‘multitrait scaling analyses’:ab,ti OR ‘item discriminant’:ab,ti OR ‘interscale correlation’:ab,ti OR ‘interscale correlations’:ab,ti OR (‘error’:ab,ti OR ‘errors’:ab,ti AND (measure*:ab,ti OR correlat*:ab,ti OR evaluat*:ab,ti OR ‘accuracy’:ab,ti OR ‘accurate’:ab,ti OR ‘precision’:ab,ti OR ‘mean’:ab,ti)) OR ‘individual variability’:ab,ti OR ‘interval variability’:ab,ti OR ‘rate variability’:ab,ti OR ‘variability analysis’:ab,ti OR (‘uncertainty’:ab,ti AND (‘measurement’:ab,ti OR ‘measuring’:ab,ti)) OR ‘standard error of measurement’:ab,ti OR sensitiv*:ab,ti OR responsive*:ab,ti OR (‘limit’:ab,ti AND ‘detection’:ab,ti) OR ‘minimal detectable concentration’:ab,ti OR

interpretab*:ab,ti OR (small*:ab,ti AND (‘real’:ab,ti OR ‘detectable’:ab,ti) AND (‘change’:ab,ti OR ‘difference’:ab,ti)) OR ‘meaningful change’:ab,ti OR ‘minimal important change’:ab,ti OR ‘minimal important difference’:ab,ti OR ‘minimally important change’:ab,ti OR ‘minimally important difference’:ab,ti OR ‘minimal detectable change’:ab,ti OR ‘minimal detectable difference’:ab,ti OR ‘minimally detectable change’:ab,ti OR ‘minimally detectable difference’:ab,ti OR ‘minimal real change’:ab,ti OR ‘minimal real difference’:ab,ti OR ‘minimally real change’:ab,ti OR ‘minimally real difference’:ab,ti OR ‘ceiling effect’:ab,ti OR ‘floor effect’:ab,ti OR ‘item response model’:ab,ti OR ‘irt’:ab,ti OR ‘rasch’:ab,ti OR ‘differential item functioning’:ab,ti OR ‘dif’:ab,ti OR ‘computer adaptive testing’:ab,ti OR ‘item bank’:ab,ti OR ‘cross-cultural equivalence’:ab,ti)

NOT

(('animal'/exp OR 'animal') NOT ('human'/exp OR 'human'))

CINAHL A
((MH"Somatosensory Disorders+") OR TI ("central sensitisation" OR "central sensitisation" OR "pain sensit*" OR "central nervous system sensitisation" OR "central nervous system sensitisation" OR "central hypersensitiv*" OR "somatosensory disorder*" OR "pain hypersensitiv*" OR "neuropathic sensitisation*" OR "neuropathic sensitisation*" OR "perceptual sensitisation*" OR "perceptual sensitisation*" OR "neural sensitisation*" OR "neural sensitisation*" OR "central sensitivity syndrome*" OR “Allodynia” OR “Hyperalgesia” OR “Hyperesthesia” OR “Hyperpathia” OR “Paresthesia” OR “Dysesthesia” OR ″Analgesia″ OR ″Hypoalgesia″ OR ″Hypoesthesia”) OR AB ("central sensitisation" OR "central sensitisation" OR "pain sensit*" OR "central nervous system sensitisation" OR "central nervous system sensitisation" OR "central hypersensitiv*" OR "somatosensory disorder*" OR "pain hypersensitiv*" OR "neuropathic sensitisation*" OR "neuropathic sensitisation*" OR "perceptual sensitisation" OR "perceptual sensitisation" OR "neural sensitisation*" OR "neural sensitisation*" OR "central sensitivity syndrome*" OR “Allodynia” OR “Hyperalgesia” OR “Hyperesthesia” OR “Hyperpathia” OR “Paresthesia” OR “Dysesthesia” OR ″Analgesia″ OR ″Hypoalgesia″ OR ″Hypoesthesia”))

AND

((MH "Low Back Pain") OR (MH "Lumbar Vertebrae") OR (MH "Radiculopathy") OR (MH "Sciatica") OR (MH "Referred Pain") OR (MH "Chronic Pain") OR TI ("low back pain*" OR "lower back pain*" OR "lumbosacral region*" OR "lumbar vertebra*" OR "radiculopathy" OR "sciatica" OR "leg pain*" OR "leg radiation*" OR "referred pain*" OR "radiating pain*" OR "radicular pain*" OR "pseudoradicular pain*" OR "lumbosacral radicular syndrome*" OR "chronic low back pain*" OR “chronic back pain*” OR “chronic pain*”) OR AB ("low back pain*" OR "lower back pain*" OR "lumbosacral region*" OR "lumbar vertebra*" OR "radiculopathy" OR "sciatica" OR "leg pain*" OR "leg radiation*" OR "referred pain*" OR "radiating pain*" OR "radicular pain*" OR "pseudoradicular pain*" OR "lumbosacral radicular syndrome*" OR "chronic low back pain*" OR “chronic back pain*” OR “chronic pain*”))

AND

((MH “Psychometrics”) or (TI psychometr* or AB psychometr*) or (TI clinimetr* or AB clinimetr*) or (TI clinometr* OR AB clinometr*) or (MH “Outcome Assessment”) or (TI outcome assessment or AB outcome assessment) or (TI outcome measure* or AB outcome measure*) or (MH “Health Status Indicators”) or (MH “Reproducibility of Results”) or (MH “Discriminant Analysis”) or ((TI reproducib* or AB reproducib*) or (TI reliab* or AB reliab*) or (TI unreliab* or AB unreliab*)) or ((TI valid* or AB valid*) or (TI coefficient or AB coefficient) or (TI homogeneity or AB homogeneity)) or (TI homogeneous or AB homogeneous) or (TI “coefficient of variation” or AB “coefficient of variation”) or (TI “internal consistency” or AB “internal consistency”) or (MH “Internal Consistency+”) or (MH “Reliability+”) or (MH

“Measurement Error+”) or (MH “Content Validity+”) or “hypothesis testing” or “structural validity” or “cross-cultural validity” or (MH “Criterion-Related Validity+”) or “responsiveness” or “interpretability” or (TI reliab* or AB reliab*) and ((TI test or AB test) OR (TI retest or AB retest)) or (TI stability or AB stability) or (TI interrater or AB interrater) or (TI inter-rater or AB inter-rater) or (TI intrarater or AB intrarater) or (TI intra-rater or AB intrarater) or (TI intertester or AB intertester) or (TI inter-tester or AB inter-tester) or (TI intratester or AB intratester) or (TI intra-tester or AB intra-tester) or (TI interobserver or AB interobserver) or (TI inter-observer or AB inter-observer) or (TI intraobserver or AB intraobserver) or (TI intra-observer or AB intra-observer) or (TI intertechnician or AB intertechnician) or (TI inter-technician or AB inter-technician) or (TI intratechnician or AB intratechnician) or (TI intra-technician or AB intra-technician) or (TI interexaminer or AB interexaminer) or (TI inter-examiner or AB inter-examiner) or (TI intraexaminer or AB intraexaminer) OR (TI intra-examiner or AB intra-examiner) or (TI intra-examiner or AB intraexaminer) or (TI interassay or AB interassay) or (TI inter-assay or AB inter-assay) or (TI intraassay or AB intraassay) or (TI intra-assay or AB intra-assay) or (TI interindividual or AB interindividual) or (TI inter-individual or AB inter-individual) OR (TI intraindividual or AB intraindividual) or (TI intra-individual or AB intra-individual) or (TI interparticipant or AB interparticipant) or (TI inter-participant or AB inter-participant) or (TI intraparticipant or AB intraparticipant) or (TI intra-participant or AB intra-participant) or (TI kappa or AB kappa) or (TI kappa's or AB kappa's) or (TI kappas or AB kappas) or (TI repeatab* or AB repeatab*) or (TI responsive* or AB responsive*) or (TI interpretab* or AB interpretab*))

NOT

((MH "Animals+") NOT(MH "Human"))

CINAHL B

((MH"Somatosensory Disorders+") OR TI ("central sensitisation" OR "central sensitisation" OR "pain sensit*" OR "central nervous system sensitisation" OR "central nervous system sensitisation" OR "central hypersensitiv*" OR "somatosensory disorder*" OR "pain hypersensitiv*" OR "neuropathic sensitisation*" OR "neuropathic sensitisation*" OR "perceptual sensitisation*" OR "perceptual sensitisation*" OR "neural sensitisation*" OR "neural sensitisation*" OR "central sensitivity syndrome*" OR “Allodynia” OR “Hyperalgesia” OR “Hyperesthesia” OR “Hyperpathia” OR “Paresthesia” OR “Dysesthesia” OR ”Analgesia” OR ”Hypoalgesia” OR ”Hypoesthesia”) OR AB ("central sensitisation" OR "central sensitisation" OR "pain sensit*" OR "central nervous system sensitisation" OR "central nervous system sensitisation" OR "central hypersensitiv*" OR "somatosensory disorder*" OR "pain hypersensitiv*" OR "neuropathic sensitisation*" OR "neuropathic sensitisation*" OR "perceptual sensitisation*" OR "perceptual sensitisation*" OR "neural sensitisation*" OR "neural sensitisation*" OR "central sensitivity syndrome*" OR “Allodynia” OR “Hyperalgesia” OR “Hyperesthesia” OR “Hyperpathia” OR “Paresthesia” OR “Dysesthesia” OR ”Analgesia” OR ”Hypoalgesia” OR ”Hypoesthesia”))

AND

((MH "Low Back Pain") OR (MH "Lumbar Vertebrae") OR (MH "Radiculopathy") OR (MH "Sciatica") OR (MH "Referred Pain") OR (MH "Chronic Pain") OR TI ("low back pain*" OR "lower back pain*" OR "lumbosacral region*" OR "lumbar vertebra*" OR "radiculopathy" OR "sciatica" OR "leg pain*" OR "leg radiation*" OR "referred pain*" OR "radiating pain*" OR "radicular pain*" OR "pseudoradicular pain*" OR "lumbosacral radicular syndrome*" OR "chronic low back pain*" OR “chronic back pain*” OR “chronic pain*”) OR AB ("low back pain*" OR "lower back pain*" OR "lumbosacral region*" OR "lumbar vertebra*" OR "radiculopathy" OR "sciatica" OR "leg pain*" OR "leg radiation*" OR "referred pain*" OR "radiating pain*" OR "radicular pain*" OR "pseudoradicular pain*" OR "lumbosacral radicular syndrome*" OR "chronic low back pain*" OR “chronic back pain*” OR “chronic pain*”))

AND

(TI psychometr* OR TI observer variation OR TI reproducib* OR TI reliab* OR TI unreliab* OR TI valid* OR TI coefficient OR TI homogeneity OR TI homogeneous OR TI "internal consistency" OR AB psychometr* OR AB observer variation OR AB reproducib* OR AB reliab* OR AB unreliab* OR AB valid* OR AB coefficient OR AB homogeneity OR AB homogeneous OR AB "internal consistency" OR (TI cronbach* OR AB cronbach* AND (TI alpha OR AB alpha OR TI alphas OR AB alphas)) OR (TI item OR AB item AND (TI correlation* OR AB correlation* OR TI selection* OR AB selection* OR TI reduction* OR AB reduction*)) OR TI agreement OR TI precision OR TI imprecision OR TI "precise values" OR TI test-retest OR AB agreement OR AB precision OR AB imprecision OR AB "precise values" OR AB test-retest OR (TI test OR AB test AND TI retest OR AB retest) OR (TI reliab* OR AB reliab* AND (TI test OR AB test OR TI retest or AB retest)) OR TI stability OR TI interrater OR TI interrater OR TI intrarater OR TI intra-rater OR TI intertester OR TI inter-tester OR TI intratester OR TI intra-tester OR TI interobserver OR TI inter-observer OR TI intraobserver OR TI intra-observer OR TI intertechnician OR TI inter-technician OR TI intratechnician OR TI intra-technician OR TI interexaminer OR TI inter-examiner OR TI intraexaminer OR TI intra-examiner OR TI interassay OR TI inter-assay OR TI intraassay OR TI intra-assay OR TI interindividual OR TI inter-individual OR TI intraindividual OR TI intra-individual OR TI interparticipant OR TI inter-participant OR TI intraparticipant OR TI intra-participant OR TI kappa OR TI kappa's OR TI kappas OR TI repeatab* OR AB stability OR AB interrater OR AB inter-rater OR AB intrarater OR AB intra-rater OR AB intertester OR AB inter-tester OR AB intratester OR AB intra-tester OR AB interobserver OR AB inter-observer OR AB intraobserver OR AB intra-observer OR AB intertechnician OR AB inter-technician OR AB intratechnician OR AB intra-technician OR AB interexaminer OR AB inter-examiner OR AB intraexaminer OR AB intra-examiner OR AB interassay OR AB inter-assay OR AB intraassay OR AB intra-assay OR AB interindividual OR AB inter-individual OR AB intraindividual OR AB intra-individual OR AB interparticipant OR AB inter-participant OR AB intraparticipant OR AB intra-participant OR AB kappa OR AB kappa's OR AB kappas OR AB repeatab* OR ((TI replicab* OR AB replicab* OR TI repeated OR AB repeated) AND (TI measure OR AB measure OR TI measures OR AB measures OR TI findings OR AB findings OR TI result OR AB result OR TI results OR AB results OR TI test OR AB test OR TI tests OR AB tests)) OR TI generaliza* OR TI generalisa* OR TI concordance OR AB generaliza* OR AB generalisa* OR AB concordance OR (TI intraclass OR AB intraclass AND TI correlation* or AB correlation*) OR TI discriminative OR TI "known group" OR TI factor analysis OR TI factor analyses OR TI dimension* OR TI subscale* OR AB discriminative OR AB "known group" OR AB factor analysis OR AB factor analyses OR AB dimension* OR AB subscale* OR (TI multitrait OR AB multitrait AND TI scaling OR AB scaling AND (TI analysis OR AB analysis OR TI analyses OR AB analyses)) OR TI item discriminant OR TI interscale correlation* OR TI error OR TI errors OR TI "individual variability" OR AB item discriminant OR AB interscale correlation* OR AB error OR AB errors OR AB "individual variability" OR (TI variability OR AB variability AND (TI analysis OR AB analysis OR TI values OR AB values)) OR (TI uncertainty OR AB uncertainty AND (TI measurement OR AB measurement OR TI measuring OR AB measuring)) OR TI "standard error of measurement" OR TI sensitiv* OR TI responsive* OR AB "standard error of measurement" OR AB sensitiv* OR AB responsive* OR ((TI minimal OR TI minimally OR TI clinical OR TI clinically OR AB minimal OR AB minimally OR AB clinical OR AB clinically) AND (TI important OR TI significant OR TI detectable OR AB important OR AB significant OR AB detectable) AND (TI change OR AB change OR TI difference OR AB difference)) OR (TI small* OR AB small* AND (TI real OR AB real OR TI detectable OR AB detectable) AND (TI change OR AB change OR TI difference OR AB difference)) OR TI meaningful change OR TI "ceiling effect" OR TI "floor effect" OR TI "Item response model" OR TI IRT OR TI Rasch OR TI "Differential item functioning" OR TI DIF OR TI "computer adaptive testing" OR TI "item bank" OR TI "cross-cultural equivalence" OR TI outcome assessment OR AB meaningful change OR AB "ceiling effect" OR AB "floor effect" OR AB "Item response model" OR AB IRT OR AB Rasch

OR AB “Differential item functioning” OR AB DIF OR AB “computer adaptive testing” OR AB “item bank” OR AB “cross-cultural equivalence” OR AB outcome assessment)

NOT

((MH "Animals+") NOT(MH "Human"))

PsycINFO A
(DE "Somatosensory Disorders" OR "central sensitisation" OR "central sensitisation" OR "pain sensit*" OR "central nervous system sensitisation" OR "central nervous system sensitisation" OR "central hypersensitivity" OR "somatosensory disorder*" OR "pain hypersensitivity" OR "neuropathic sensitisation" OR "neuropathic sensitisation" OR "perceptual sensitisation" OR "perceptual sensitisation" OR "neural sensitisation*" OR "neural sensitisation*" OR "central sensitivity syndrome*" OR “Allodynia” OR “Hyperalgesia” OR “Hyperesthesia” OR “Hyperpathia” OR “Paresthesia” OR “Dysesthesia” OR ”Analgesia” OR ”Hypoalgesia” OR ”Hypoesthesia”)

AND

(DE "Lumbar Spinal Cord" OR DE "Chronic Pain" OR DE "Back Pain" OR "low back pain*" OR "lower back pain*" OR "lumbarsacral region" OR "lumbar vertebra*" OR "radiculopathy" OR "sciatica" OR "leg pain" OR "leg radiation" OR "referred pain" OR "radiating pain" OR "radicular pain" OR "pseudoradicular pain" OR "lumbosacral radicular syndrome" OR "chronic low back pain" OR "lumbar spinal cord")

AND

(TI psychometr* OR TI observer variation OR TI reproducib* OR TI reliab* OR TI unreliab* OR TI valid* OR TI coefficient OR TI homogeneity OR TI homogeneous OR TI “internal consistency” OR AB psychometr* OR AB observer variation OR AB reproducib* OR AB reliab* OR AB unreliab* OR AB valid* OR AB coefficient OR AB homogeneity OR AB homogeneous OR AB “internal consistency” OR (TI cronbach* OR AB cronbach* AND (TI alpha OR AB alpha OR TI alphas OR AB alphas)) OR (TI item OR AB item AND (TI correlation* OR AB correlation* OR TI selection* OR AB selection* OR TI reduction* OR AB reduction*)) OR TI agreement OR TI precision OR TI imprecision OR TI “precise values” OR TI test-retest OR AB agreement OR AB precision OR AB imprecision OR AB “precise values” OR AB test-retest OR (TI test OR AB test AND TI retest OR AB retest) OR (TI reliab* OR AB reliab* AND (TI test OR AB test OR TI retest or AB retest)) OR TI stability OR TI interrater OR TI inter-rater OR TI intrarater OR TI intra-rater OR TI intertester OR TI inter-tester OR TI intratester OR TI intra-tester OR TI interobserver OR TI inter-observer OR TI intraobserver OR TI intra-observer OR TI intertechnician OR TI inter-technician OR TI intratechnician OR TI intra-technician OR TI interexaminer OR TI inter-examiner OR TI intraexaminer OR TI intra-examiner OR TI interassay OR TI inter-assay OR TI intraassay OR TI intra-assay OR TI interindividual OR TI inter-individual OR TI intraindividual OR TI intra-individual OR TI interparticipant OR TI inter-participant OR TI intraparticipant OR TI intra-participant OR TI kappa OR TI kappa’s OR TI kappas OR TI repeatab* OR AB stability OR AB interrater OR AB inter-rater OR AB intrarater OR AB intra-rater OR AB intertester OR AB inter-tester OR AB intratester OR AB intra-tester OR AB interobserver OR AB inter-observer OR AB intraobserver OR AB intra-observer OR AB intertechnician OR AB inter-technician OR AB intratechnician OR AB intra-technician OR AB interexaminer OR AB inter-examiner OR AB intraexaminer OR AB intra-examiner OR AB interassay OR AB inter-assay OR AB intraassay OR AB intra-assay OR AB interindividual OR AB inter-individual OR AB intraindividual OR AB intra-individual OR AB interparticipant OR AB inter-participant OR AB intraparticipant OR AB intra-participant OR AB kappa OR AB kappa’s OR AB kappas OR AB repeatab* OR ((TI replicab* OR AB replicab* OR TI repeated OR AB repeated) AND (TI measure OR AB measure OR TI measures OR AB

measures OR TI findings OR AB findings OR TI result OR AB result OR TI results OR AB results OR TI test OR AB test OR TI tests OR AB tests)) OR TI generaliza* OR TI generalisa* OR TI concordance OR AB generaliza* OR AB generalisa* OR AB concordance OR (TI intraclass OR AB intraclass AND TI correlation* or AB correlation*) OR TI discriminative OR TI "known group" OR TI factor analysis OR TI factor analyses OR TI dimension* OR TI subscale* OR AB discriminative OR AB "known group" OR AB factor analysis OR AB factor analyses OR AB dimension* OR AB subscale* OR (TI multitrait OR AB multitrait AND TI scaling OR AB scaling AND (TI analysis OR AB analysis OR TI analyses OR AB analyses)) OR TI item discriminant OR TI interscale correlation* OR TI error OR TI errors OR TI "individual variability" OR AB item discriminant OR AB interscale correlation* OR AB error OR AB errors OR AB "individual variability" OR (TI variability OR AB variability AND (TI analysis OR AB analysis OR TI values OR AB values)) OR (TI uncertainty OR AB uncertainty AND (TI measurement OR AB measurement OR TI measuring OR AB measuring)) OR TI "standard error of measurement" OR TI sensitiv* OR TI responsive* OR AB "standard error of measurement" OR AB sensitiv* OR AB responsive* OR ((TI minimal OR TI minimally OR TI clinical OR TI clinically OR AB minimal OR AB minimally OR AB clinical OR AB clinically) AND (TI important OR TI significant OR TI detectable OR AB important OR AB significant OR AB detectable) AND (TI change OR AB change OR TI difference OR AB difference)) OR (TI small* OR AB small* AND (TI real OR AB real OR TI detectable OR AB detectable) AND (TI change OR AB change OR TI difference OR AB difference)) OR TI meaningful change OR TI "ceiling effect" OR TI "floor effect" OR TI "Item response model" OR TI IRT OR TI Rasch OR TI "Differential item functioning" OR TI DIF OR TI "computer adaptive testing" OR TI "item bank" OR TI "cross-cultural equivalence" OR TI outcome assessment OR AB meaningful change OR AB "ceiling effect" OR AB "floor effect" OR AB "Item response model" OR AB IRT OR AB Rasch OR AB "Differential item functioning" OR AB DIF OR AB "computer adaptive testing" OR AB "item bank" OR AB "cross-cultural equivalence" OR AB outcome assessment)

PsycINFO B
(DE "Somatosensory Disorders" OR "central sensitisation" OR "central sensitisation" OR "pain sensit*" OR "central nervous system sensitisation" OR "central nervous system sensitisation" OR "central hypersensitiv*" OR "somatosensory disorder*" OR "pain hypersensitiv*" OR "neuropathic sensitisation*" OR "neuropathic sensitisation*" OR "perceptual sensitisation*" OR "perceptual sensitisation*" OR "neural sensitisation*" OR "neural sensitisation*" OR "central sensitivity syndrome*" OR "Allodynia" OR "Hyperalgesia" OR "Hyperesthesia" OR "Hyperpathia" OR "Paresthesia" OR "Dysesthesia" OR "Analgesia" OR "Hypoalgesia" OR "Hypoesthesia")

AND

(DE "Lumbar Spinal Cord" OR DE "Chronic Pain" OR DE "Back Pain" OR "low back pain*" OR "lower back pain*" OR "lumbarsacral region*" OR "lumbar vertebra*" OR "radiculopathy" OR "sciatica" OR "leg pain*" OR "leg radiation*" OR "referred pain*" OR "radiating pain*" OR "radicular pain*" OR "pseudoradicular pain*" OR "lumbosacral radicular syndrome*" OR "chronic low back pain*" OR "lumbar spinal cord*" OR "chronic back pain*" OR "chronic pain*")

AND

cl("Psychometrics & Statistics & Methodology" OR "Research Methods & Experimental Design") OR (psychometr* OR clinimetr* OR clinometr* OR "outcome assessment" OR "outcome measure*" OR "observer variation" OR reproducib* OR reliab* OR unreliab* OR valid* OR coefficient OR homogeneity OR homogeneous OR "internal consistency" OR agreement OR precision OR imprecision OR "precise values" OR test-retest OR reliab* OR stability OR interrater OR inter-rater OR intrarater OR intra-rater OR intertester OR inter-tester OR intratester OR intra-tester OR interobserver OR inter-observer OR intraobserver OR intra-observer OR intertechnician OR inter-technician OR intratechnician OR

intra-technician OR interexaminer OR inter-examiner OR intraexaminer OR intra-examiner OR interassay OR inter-assay OR intraassay OR intra-assay OR interindividual OR inter-individual OR intraindividual OR intra-individual OR interparticipant OR inter-participant OR intraparticipant OR intra-participant OR kappa OR kappa's OR kappas OR repeatab* OR generaliza* OR generalisa* OR concordance OR discriminative OR "known group" OR "factor analys*" OR dimension* OR subscale* OR "item discriminant" OR "interscale correlation*" OR error* OR "individual variability" OR "standard error of measurement" OR sensitiv* OR responsive* OR "meaningful change" OR "ceiling effect" OR "floor effect" OR "Item response model" OR IRT OR Rasch OR "Differential item functioning" OR DIF OR "computer adaptive testing" OR "item bank" OR "cross-cultural equivalence") OR ("cronbach* alpha*" OR "replicab* measure*" OR "replicab* finding*" OR "replicab* result*" OR "replicab* test*" OR "repeated measure*" OR "repeated finding*" OR "repeated result*" OR "repeated test*" OR "item correlation*" OR "item selection*" OR "item reduction*" OR "Test retest" OR "intraclass correlation*" OR "multitrait scaling analys*" OR "uncertainty measur*" OR "variability analys*" OR "variability value*" OR "minimal* important change" OR "minimal* important difference" OR "minimal* significant change" OR "minimal* significant difference" OR "minimal* detectable change" OR "minimal* detectable difference" OR "clinical* important change" OR "clinical* important difference" OR "clinical* significant change" OR "clinical* significant difference" OR "clinical* detectable change" OR "clinical* detectable difference" OR "small* real change" OR "small* real difference" OR "small* detectable change" OR "small* detectable difference") OR (SU.EXACT.EXPLODE("Measurement") OR SU.EXACT.EXPLODE("Error Analysis") OR SU.EXACT.EXPLODE("Test Construction") OR SU.EXACT.EXPLODE("Interrater Reliability") OR SU.EXACT.EXPLODE("Content Analysis") OR SU.EXACT.EXPLODE("Error of Measurement") OR SU.EXACT.EXPLODE("Factor Structure") OR SU.EXACT.EXPLODE("Testing Methods") OR SU.EXACT.EXPLODE("Statistical Reliability") OR SU.EXACT.EXPLODE("Consistency (Measurement)") OR SU.EXACT.EXPLODE("Computer Assisted Testing") OR SU.EXACT.EXPLODE("Factor Analysis") OR SU.EXACT.EXPLODE("Prediction") OR SU.EXACT.EXPLODE("Statistical Validity") OR SU.EXACT.EXPLODE("Prediction Errors"))

Table S1. Signalling Questions Quadas-2

	Risk of Bias	Patient Selection
Patient selection	*Was a consecutive or random sample of patients enrolled?*<br>*Was a case-control design avoided?*<br>*Did the study avoid inappropriate exclusions?*<br>**Could the selection of patients have introduced bias?**	**Is there concern that the included patients do not match the review question?**
Index Test(s)	*Were the index test results interpreted without knowledge of the results of the reference standard?*<br>*If a threshold was used, was it pre-specified?*<br>**Could the conduct or interpretation of the index test have introduced bias?**	**Is there concern that the index test, its conduct, or interpretation differ from the review question?**
Reference standard	*Is the reference standard likely to correctly classify the target condition?*<br>*Were the reference standard results interpreted without knowledge of the results of the index test?*<br>**Could the reference standard, its conduct, or its interpretation have introduced bias?**	**Is there concern that the target condition as defined by the reference standard does not match the review question?**
Flow and timing	*Was there an appropriate interval between index test(s) and reference standard?*<br>*Did all patients receive a reference standard?*<br>*Did patients receive the same reference standard?*<br>*Were all patients included in the analysis?*<br>**Could the patient flow have introduced bias?**	

Legend: The italics signalling questions are sub signalling questions to help answer the bold signalling question. Adapted from Whiting et al., 2011 [37, 38].

Table S2. Correlations of the Measures Used to Assess HACS to Other Measures

				solely CLBP	CLBP+	solely CLBP	solely CLBP	CLBP
1st Author, year		Test	Type	CSI part A		MBM	MPQ	WPI
Huysmans, 2018	[43]	1-minute stair-climbing test	PA		-0.34			
Noord van der, 2018	[85]	Anxiety (SCL-90)	Q	0.65				
Miki, 2020	[73]	Anxiety: Hospital Anxiety and Depression Scale	Q		0.50			
Huysmans, 2018	[43]	Brief Illness Perception Questionnaire	Q		0.40			
Noord van der, 2018	[85]	Central sensitivity syndrome	Q		0.51			
Kregel, 2016	[56]	Conditioned Pain Modulation	PA		0.02			
Aoyagi,2019	[7]	Conditioned Pain Modulation: Lower Back	PA					-0.24
Aoyagi,2019	[7]	Conditioned Pain Modulation: Thumbnail	PA					-0.49
Serrano-Ibáñez, 2020#	[99]	Daily routines	Q		-0.28			
Serrano-Ibáñez, 2020#	[99]	Decreased physical activity	Q		0.29			
Noord van der, 2018	[85]	Depression (SCL-90)	Q	0.67				
Miki, 2020	[73]	Depression: Hospital Anxiety and Depression Scale	Q		0.49			
Serrano-Ibáñez, 2020#	[99]	Diminished social support	Q		0.49			
Mibu, 2019	[71]	Duration symptoms	Q		0.02			
Serrano-Ibáñez, 2020#	[99]	Emotional distress	Q		0.56			
Ide, 2020#	[44]	Euro QoL 5 Dimensions	Q	-0.41	-0.42			
Mibu, 2019	[71]	Euro QoL 5 Dimensions	Q		-0.47			
Miki, 2020	[73]	Euro QoL 5 Dimensions	Q		-0.37			
Knezevic, 2020#	[51]	Fear-Avoidance Component Scale: factor 1	Q	0.43	0.29			
Knezevic, 2020#	[51]	Fear-Avoidance Component Scale: factor 2	Q	0.24	0.18			
Knezevic, 2020#	[51]	Fear-Avoidance Component Scale: total score	Q	0.41	0.28			
Ansuategui Echeita, 2020b	[6]	Lifting capacity	PA		-0.53			
McKernan, 2019	[64]	McGill Pain Questionnaire	Q	0.62		0.47		
Sharma, 2020	[100]	McGill Pain Questionnaire	Q		0.35			
McKernan, 2019	[64]	MEAQ subscore: behavioral avoidance	Q	0.34		0.14	0.26	
McKernan, 2019	[64]	MEAQ subscore: Distraction and suppression	Q	0.34		0.23	0.38	
McKernan, 2019	[64]	MEAQ subscore: Distress aversion	Q	0.33		0.18	0.30	
McKernan, 2019	[64]	MEAQ subscore: Distress endurance	Q	-0.22		-0.02	-0.09	
McKernan, 2019	[64]	MEAQ subscore: Procrastination	Q	0.37		0.17	0.24	

				solely CLBP	CLBP+	solely CLBP	solely CLBP	CLBP
1st Author, year		Test	Type	CSI part A		MBM	MPQ	WPI
McKernan, 2019	[64]	MEAQ subscore: Repression and denial	Q	0.20		0.09	0.22	
Knezevic, 2020#	[51]	Mental component (Short Form-36)	Q	-0.40	-0.36			
Kregel, 2018	[55]	Mental component (Short Form-36)	Q	-0.64				
McKernan, 2019	[64]	Michigan Body Map	Q	0.55				
Knezevic, 2020#	[51]	MOS cognitive functioning scale: Medical Outcomes Study	Q	-0.44	-0.43			
McKernan, 2019	[64]	Multidimensional Experiential Avoidance Questionnaire (MEAQ) total score	Q	0.42		0.22	0.36	
Knezevic, 2020#	[51]	Multidimentional Scale of Perceived Social Support (MSPSS); total score	Q	-0.18	-0.12			
Knezevic, 2020#	[51]	Multidimentional Scale of Perceived Social Support: family	Q	-0.15	-0.10			
Knezevic, 2020#	[51]	Multidimentional Scale of Perceived Social Support: friends	Q	-0.20	-0.12			
Knezevic, 2020#	[51]	Multidimentional Scale of Perceived Social Support: significant other	Q	-0.14	-0.09			
Ide, 2020#	[44]	Neck Disability Index	Q	0.58	0.60			
Ide, 2020#	[44]	Oswestry Disability Index	Q	0.42	0.50			
Knezevic, 2020#	[51]	Oswestry Disability Index	Q	0.34	0.30			
Bilika, 2019#	[15]	Pain catastrophizing Scale	Q	0.74	0.56			
Huysmans, 2018	[43]	Pain catastrophizing Scale	Q	0.52				
Knezevic, 2020#	[51]	Pain catastrophizing Scale	Q	0.38	0.24			
Kregel, 2018	[55]	Pain catastrophizing Scale	Q	0.46				
Miki, 2020	[73]	Pain catastrophizing Scale	Q	0.54				
Noord van der, 2018	[85]	Pain catastrophizing Scale	Q	0.29				
Sharma, 2020	[100]	Pain catastrophizing Scale	Q	0.50				
Kregel, 2018	[55]	Pain disability index	Q	0.47				
Sharma, 2020	[100]	Pain duration	Q	0.10				
Miki, 2020	[73]	Pain Score: NRS for leg pain	Q	0.21				
Ide, 2020	[44]	Pain Score: NRS for low back pain	Q	0.37				
Miki, 2020	[73]	Pain Score: NRS for low back pain	Q	0.28				
Ide, 2020	[44]	Pain Score: NRS for lower limb	Q	0.43				
Ide, 2020	[44]	Pain Score: NRS for neck pain	Q	0.43				
Ide, 2020	[44]	Pain Score: NRS for upper limb	Q	0.49				
Mibu, 2019	[71]	Pain Score: NRS interference	Q	0.42				

1st Author, year		Test	Type	solely CLBP CSI part A	CLBP+	solely CLBP MBM	solely CLBP MPQ	CLBP WPI
Kregel, 2018	[55]	Pain Score: NRS pain intensity	Q	0.32				
Mibu, 2019	[71]	Pain Score: NRS pain intensity	Q	0.37				
Noord van der, 2018	[85]	Pain Score: NRS pain intensity	Q	0.36				
Serrano-Ibáñez, 2020#	[99]	Pain Score: NRS pain intensity	Q	0.60				
Sharma, 2020	[100]	Pain Score: NRS pain intensity	Q	0.25				
Knezevic, 2020#	[51]	Pain Score: NRS pain intensity: Average pain past 4 weeks	Q	0.30	0.10			
Knezevic, 2020#	[51]	Pain Score: NRS pain intensity: now	Q	0.27	-0.02			
Knezevic, 2020#	[51]	Pain Score: NRS pain intensity: Strongest pain past 4 weeks	Q	0.28	0.06			
Huysmans, 2018	[43]	Pain Score: VAS: 7 days	Q	0.51				
Huysmans, 2018	[43]	Pain Score: VAS: now	Q	0.51				
McKernan, 2019	[64]	PTSD (PCL)	Q	0.65		0.32	0.51	
Knezevic, 2020#	[51]	PCS helplessness subscale	Q	0.40	0.29			
Knezevic, 2020#	[51]	PCS magnification subscale	Q	0.36	0.25			
Knezevic, 2020#	[51]	PCS rumination subscale	Q	0.31	0.12			
Knezevic, 2020#	[51]	Physical component (Short Form-36)	Q	-0.25	-0.20			
Kregel, 2018	[55]	Physical component (Short Form-36)	Q	-0.62				
Mibu, 2019	[71]	Pressure Pain Threshold	PA	-0.09				
Kregel, 2018	[55]	Pressure Pain Threshold: leg	PA	-0.28				
Aoyagi,2019	[7]	Pressure Pain Threshold: Lower Back	PA					-0.44
Kregel, 2018	[55]	Pressure Pain Threshold: Lower Back	PA	-0.26				
Aoyagi,2019	[7]	Pressure Pain Threshold: Thumbnail	PA					-0.30
Kregel, 2018	[55]	Pressure Pain Threshold: trapezius	PA	-0.24				
Kregel, 2018	[55]	Pressure Pain Threshold:] hand	PA	-0.26				
Huysmans, 2018	[43]	Quebec Back Pain Disability Scale	Q	0.40				
Miki, 2020	[73]	Roland Morris Disability Questionnaire	Q	0.28				
Clark, 2019	[25]	Sensory profile: Sensation Avoidance	Q	0.48				
Clark, 2018	[26]	Sensory profile: Low Registration	Q	none				
Clark, 2019	[25]	Sensory profile: Low Registration	Q	0.54				
Clark, 2018	[26]	Sensory profile: Sensation Avoidance	Q	none				

				solely CLBP	CLBP+	solely CLBP	solely CLBP	CLBP
**1st Author, year**		**Test**	**Type**	**CSI part A**		**MBM**	**MPQ**	**WPI**
Clark, 2018	[26]	Sensory Profile: Sensory seeking	Q	-0.53				
Clark, 2019	[25]	Sensory Profile: Sensory seeking	Q	-0.23				
Clark, 2018	[26]	Sensory Profile: Sensory Sensitive	Q	0.57				
Clark, 2019	[25]	Sensory Profile: Sensory Sensitive	Q	0.63				
Knezevic, 2020#	[51]	Sleep problem Index II (MOS sleep scale)	Q	-0.52	-0.47			
Huysmans, 2018	[43]	Tampa Scale for Kinesiophobia	Q	0.35				
Miki, 2020	[73]	Tampa Scale for Kinesiophobia	Q	0.26				
Mibu, 2019	[71]	Temporal summation	PA	0.09				
Clark, 2018	[26]	The State-Trait Anxiety Inventory	Q	0.63				
Clark, 2019	[25]	The State-Trait Anxiety Inventory	Q	0.44				
McKernan, 2019	[64]	Trauma History Questionnaire	Q	0.28		0.32	0.28	
Ansuategui Echeita, 2020a	[5]	Waddle Non-organic Signs	PA	0.34				
Mibu, 2019	[71]	Widespread Pain Index	Q	0.25				
Noord van der, 2018	[85]	Widespread Pain Index	Q	0.43				

Legend: [light blue] : positive values; [dark blue] : negative values, CLBP: chronic low back pain, CLBP+: chronic low back pain in combination with other pain condition(s), CPM: conditioned pain modulation, CSI A: Central sensitisation inventory part A, MBM: Michigan body map, MOS: medical outcomes study, MPQ: McGill pain questionnaire, MSPSS: multidimensional scale of perceived social support, NRS: Numeric rating scale, PCS: pain catastrophising scale, PPT: pain pressure threshold, SCL-90: Symptom checklist, VAS: visual analogue scale, WPI: Widespread pain index. # Data provided by authors'.
